# Plasmonic Nanowires for Wide Wavelength Range Molecular Sensing

**DOI:** 10.3390/ma11050827

**Published:** 2018-05-17

**Authors:** Giovanni Marinaro, Gobind Das, Andrea Giugni, Marco Allione, Bruno Torre, Patrizio Candeloro, Jurgen Kosel, Enzo Di Fabrizio

**Affiliations:** 1 Division of Computer, Electrical and Mathematical Sciences and Engineering (CEMSE), King Abdullah University of Science and Technology (KAUST), Thuwal 23955-6900, Saudi Arabia; giovanni.marinaro@kaust.edu.sa (G.M.); jurgen.kosel@kaust.edu.sa (J.K.); 2 Structural Molecular Imaging Light Enhanced Spectroscopies Laboratory, Physical Science and Engineering, Division, King Abdullah University of Science and Technology (KAUST), Thuwal 23955-6900, Saudi Arabia; gobind.das@kaust.edu.sa (G.D.); andrea.giugni@kaust.edu.sa (A.G.); marco.allione@kaust.edu.sa (M.A.); bruno.torre@kaust.edu.sa (B.T.); 3 Bio Nano Mechanical Laboratory, Department of Experimental and Clinical Medicine, University Magna Graecia, 88100 Catanzaro, Italy; patrizio.candeloro@unicz.it

**Keywords:** surface-enhanced Raman spectroscopy, nanowires, plasmon resonance

## Abstract

In this paper, we propose the use of a standing nanowires array, constituted by plasmonic active gold wires grown on iron disks, and partially immersed in a supporting alumina matrix, for surface-enhanced Raman spectroscopy applications. The galvanic process was used to fabricate nanowires in pores of anodized alumina template, making this device cost-effective. This fabrication method allows for the selection of size, diameter, and spatial arrangement of nanowires. The proposed device, thanks to a detailed design analysis, demonstrates a broadband plasmonic enhancement effect useful for many standard excitation wavelengths in the visible and NIR. The trigonal pores arrangement gives an efficiency weakly dependent on polarization. The devices, tested with 633 and 830 nm laser lines, show a significant Raman enhancement factor, up to around 6 × 10^4^, with respect to the flat gold surface, used as a reference for the measurements of the investigated molecules.

## 1. Introduction

Surface enhanced Raman scattering (SERS), one of the elected techniques in material science, as well as in biological investigation, for low molecular concentration detection and chemical bond analysis, has been greatly developed, demonstrating a sensitivity down to single molecule detection [1,2]. By using SERS, it is possible to detect molecules adsorbed or lying in the proximity of the surface of metallic nanostructures, where a giant and strongly confined electric field (hot spots) is superimposed to a chemical SERS [3]. 

A large amount of quite different SERS substrates has been already proposed, ranging from simply chemically roughened metallic surfaces to patterned nanostructures. Plasmonic structures, as diverse as nano-spheres, -antennas, -cuboids, -holes, -triangles, honeycombs, etc., have been demonstrated to be a viable and effective solution. In all those systems, the aim is to obtain electrical near field localization at subwavelength scales. This can be achieved either with *top-down* or with *bottom-up* fabrication approaches. The enhancement is due to localized surface plasmon resonance (LSPR) of a single or of an interacting array of nanostructures [4,5]. The LSPR depends on size, shape, dielectric constants of the surrounding materials, and on array geometry and interparticle gap distances. The fine-tuning of all these parameters contributes to a significant increase in the scattered Raman signal [6,7]. Furthermore, SERS has a unique advantage over other absorption vibrational spectroscopic techniques because of its suitability in an aqueous environment [8]. 

The *top-down* approach, involving techniques like e-beam or optical lithography, enables the reliable and reproducible fabrication of devices with high enhancement factors, whereas the *bottom-up* approach, mainly based on the synthesis of nanoparticles by means of chemical technique, has the advantage of the high throughput of device production at a very low cost [9,10,11,12,13,14,15,16,17,18,19]. As an example of the first method, researchers recently reported the fabrication by e-beam lithography of 3D-plasmonic devices with giant enhancement, allowing the detection down to single molecule level [7,20,21]. On the other hand, differently shaped nanoparticles (nano-stars, -octahedral, etc.) were synthesized by means of chemical technique, leading to devices which are suitable to detect low concentrations in solution. All devices reported so far, realized with both approaches, show some limitations: those relying on very sophisticated fabrication techniques, such as e-beam or optical lithography, are typically expensive, and limit the size of the final active area to few micron squares, hindering their *scaling up* and broad application in routine analytical laboratories [19,22]. On the other hand, the structures obtained by chemical synthesis show a lack of precise spatial control of the grown nanostructures.

Some combined techniques have been recently proposed, like the use of large-area interference optical lithography, providing an interesting alternative to the two mainstream approaches. Nevertheless, the deposition of the nanostructures, in this case, was made by high-vacuum evaporation [18], implying higher costs with respect to standard *bottom-up* techniques and, more importantly, resulting in pretty large interstructure gaps that limit the enhancement..

This work, based on electrochemical deposition, allows low-cost, large-scale and controlled spatial deposition, with intergap distance at the nanoscale. In previous works, we used e-beam lithography in combination with the electroless deposition of silver, leading again to very high resolution structures, but limited by cost and surface coverage [23]. Anodized porous alumina (APA) is a very attractive fabrication method, as it can be realized by simple anodization in liquid solution on large areas, with the possibility to control structural parameters, like pore size and intergap distance. After electrochemical deposition, upon removal of alumina, we get very small and controlled gaps between the grown structures [24]. Moreover, the native trigonal layout of the pores in this material ensures the highest possible density of cylindrical structures per unit area, and consequently, results in the highest possible density of gaps. Since the hot spots are located in the gaps of the structure, a high density of gaps is certainly desirable to maximize the final device enhancement and sensitivity.

In this paper, we investigate the fabrication of forest-type gold nanowire SERS substrates by means of an optimized electrochemical technique, realizing them in a fast and cost-effective process with a large active area of cm^2^ range [25,26]. The novelty of this work is to attain good control and reproducibility in having the forest-type gold-based SERS substrate, down to 6 nm gap. The fabrication process involves the electrochemical etching and subsequent noble metal (gold, in this case) electroplating growth [26,27]. 

Below, we report the fabrication of different substrates, the numerical simulation study, and the optical characterizations of the device. Preliminary spectroscopic tests were carried out with chemisorbed rhodamine 6G (R6G) and benzenethiol (BZT) molecules. We choose these two different molecules, one fluorescent, and the other a thiolated molecule, because they are conventionally used as SERS benchmarks. The experiments showed a high enhancement of Raman signal when compared to a reference sample, with the best results for excitation at 830 nm. SERS enhancement factor was found to be around 1.6 × 10^4^ with respect to an evaporated Au surface, with an average surface roughness of 5 nm (which itself carries a Raman enhancement in the range of 10).

We evaluated field enhancement factors by means of finite-difference time-domain (FDTD) simulations. We modelled our plasmonic devices in order to reconstruct, in detail, the electric field distribution when excited by different linear polarizations. Various calculations were performed on different devices with varying wire diameters and gaps. The far-field extinction spectra show the wide resonance band for these devices. In addition, near-field plasmonic behavior shows the localization of electric fields in the gap between nanowires, consistent to the experimental findings in terms of enhancement factors.

Finally, we also report the density functional theory (DFT) calculation for BZT in a simplified scheme where a single BZT molecule has bonded to a gold atom. We compared the results with the experimental findings.

## 2. Results

**Figure 1:**
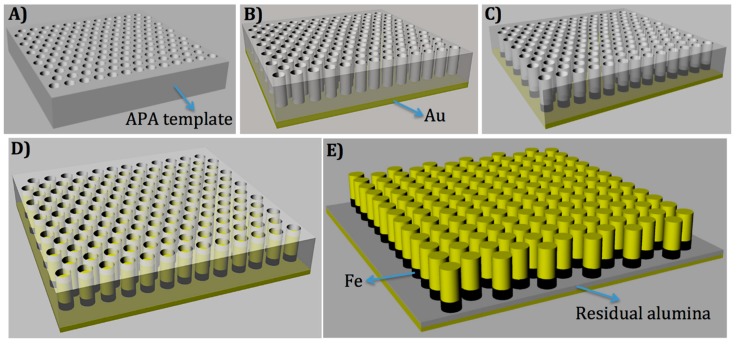
Schematic diagram of forest-type nanowire fabrication. (**A**) Anodic porous alumina (APA) template; (**B**) after gold evaporation on the backside of pores and etching out of aluminum layer; (**C**) after the electrodeposition of Fe in the APA pores; (**D**) after the electrodeposition of Au pores; and (**E**) after partial etching of alumina (residual layer of alumina), forming the final SERS device with Au nanowires (on top).

**Figure 2:**
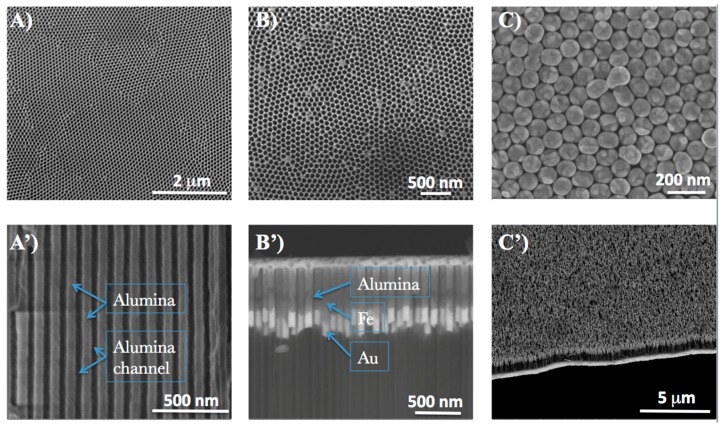
SEM images of different samples at different fabrication steps: (**A**,**A’**) APA template fabricated from the aluminum disc; (**B**,**B’**) after electrodeposition of metals in the APA channels with Fe and Au in the column; (**C**,**C’**) the forest-type Au (400 nm)/Fe nanowires after wet-etching of alumina.

Forest-structured gold nanowires were fabricated using electrochemical technique after iron electrodeposition. We describe the process flow for SERS device fabrication in Figure 1. Process scheme of anodic porous alumina (APA) template, electroplating growth of Fe and Au, and then the etching result of APA (formation of nanowires) top view, are shown in Figure 2A–C. The side view is shown in Figure 2A’–C’. An APA template with a pore size of 40 nm, and the wall thickness in the range of 10–50 nm was, fabricated in a large surface area (about 10 mm diameter) [16,28,29]. The APA pore channels can be observed in Figure 2A’. After etching, the electrodeposition of Fe and Au followed. The height of the fabricated samples spans in the range of 50–100 nm for Fe and 250–400 nm for Au. Various samples were further prepared by varying the APA channel width to attain wire gaps as small as 2–6 nm (APA wall thickness). In the final stage, porous alumina wall was partially etched out to attain higher mechanical stability for the gold nanowires [30,31]. The fabrication process is described in the Materials and Methods section. 

**Figure 3:**
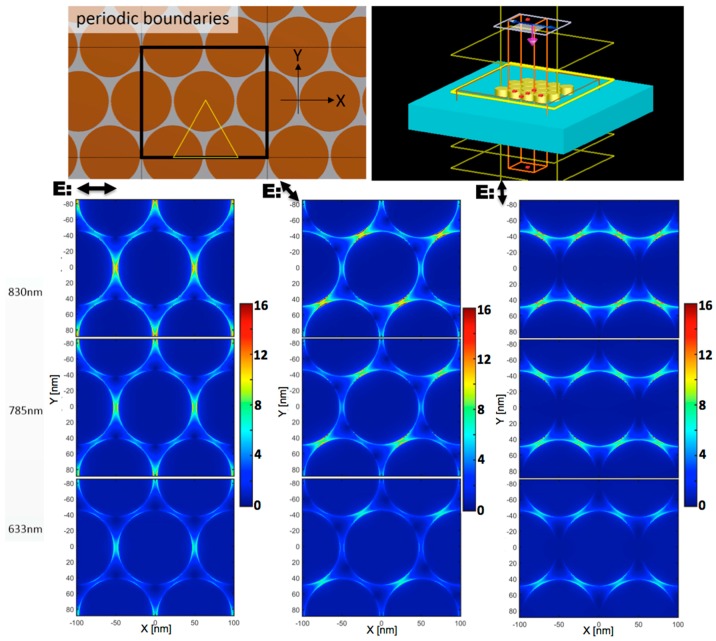
Electromagnetic FDTD simulations of the device. In the upper panels, the design of plasmonic device superimposed by the periodic boundaries (top view) and the whole simulation volume with monitors and source (perspective view). In the bottom panels, the electric field amplitude distribution (normalized to the excitation) obtained at 633, 785, and 830 nm when excited with plane wave, *in plane* polarized along the short wire interaxis (horizontal), at 60 degrees, and along the long interaxis (vertical).

A relevant part of this paper is dedicated to the detailed study of the electromagnetic behavior of the fabricated devices. To investigate the electric field distribution in our gold-based nanowire array, we performed FDTD numerical simulations using a commercial software [32]. The sample is illuminated by a linearly polarized plane wave in the XY-plane, propagating with its wave vector orthogonal to the surface. We studied nanowires partially immersed in the APA substrate channels, organized on a trigonal lattice structure defining the XY-plane of the simulation. We adopted infinite periodic boundary conditions. We set a 0.5 nm mesh resolution on the XY plane, and 1 nm along the Z direction. The structure parameters were retrieved from the sample used for the Raman experiment. In particular, the height, the diameter, and the intergap of Au nanowires reported in the simulations were set to 400, 97, and 6 nm, respectively. Figure 3 shows the design of the plasmonic device (top panel), and the electric field distribution at 633, 785, and 830 nm (bottom panel). We report the electric field amplitude spatial distribution for the excitation at 405, 570, 633, 785, and 830 nm wavelengths, aside the full spectral response of the device as a function of the intergap position in Supplementary Information S1. To calculate the field distribution, we chose, after symmetry consideration, three different representative *in plane* polarizations of incident light, as indicated in Figure 3: along X-axis, along Y-axis, and 60° from X-axis. The simulations show that the confinement of electric field remains strong for the wavelength range from 550 nm to the NIR. They show how it appears as a double contribution to the field enhancement, one from single nanowire at the high-frequency edge, at about 570 nm, and a second broad structured term from the periodic array of vertical nanowires, present from visible to near infrared wavelength (see Supplementary S1). We remark that the local maxima of the electric fields remain invariant, independently of the excitation polarization investigated: they are stronger near the metal tops along the interwire axis and characterized by a specific spectral shape. The electric field amplitude is about two times higher, when the structure is excited at 830 nm, than the excitation is at 633 nm. In Supplementary Information Figure S1a–e, we also report the spectral electric field amplitude at different positions within the gap as a function of the field polarization direction, and for different vertical distances, below and above the nanowire tops. We also simulated the single wire case, as shown in Figure S1h, in order to identify its contribution to the total field peak enhancement around 570 nm. The LSPR of the single nanowire can be seen in Figure S1f,g. As part of the optical characterization of the devices, we also evaluated the transmittance and reflectance of three different cases: SERS device, device with a single standing wire, and alumina thin film. Figure S1h evidences the effect of array periodicity in the reflected pattern, while the contribution of single wire became evident, compared to the simple alumina layer. From Figure S1d, we get as a general trend that the electric field spectral profile near the gaps shows a broad enhancement region, having its maximum in the NIR. We also got the effect of the gap width on the device performances from a specific FDTD simulation set. Figure S1i reports the spectra obtained, in this case, at the edge of the wire and at the center of the gap for structures having different gap widths, from 3 to 20 nm, and the same periodicity. As the figure shows, the highest field enhancement is observed in the structure having the narrower gap, about a factor 3 larger than the 20 nm case.

**Figure 4:**
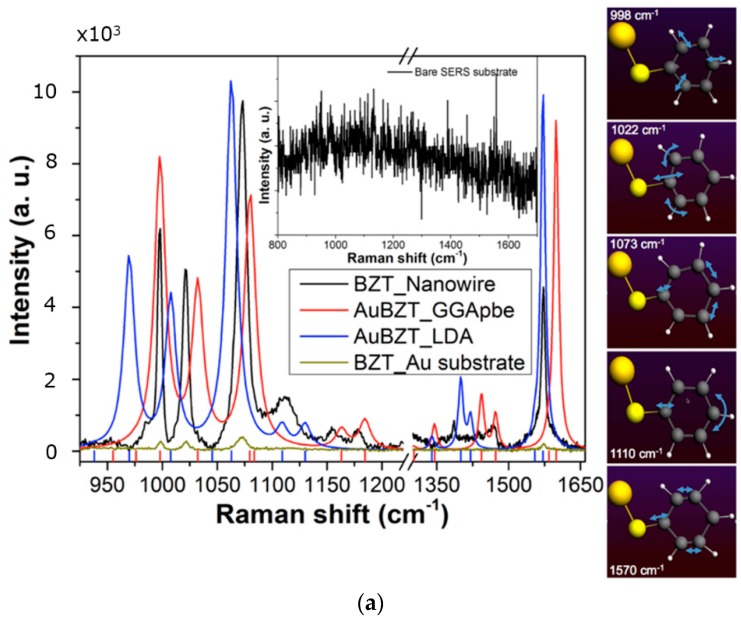

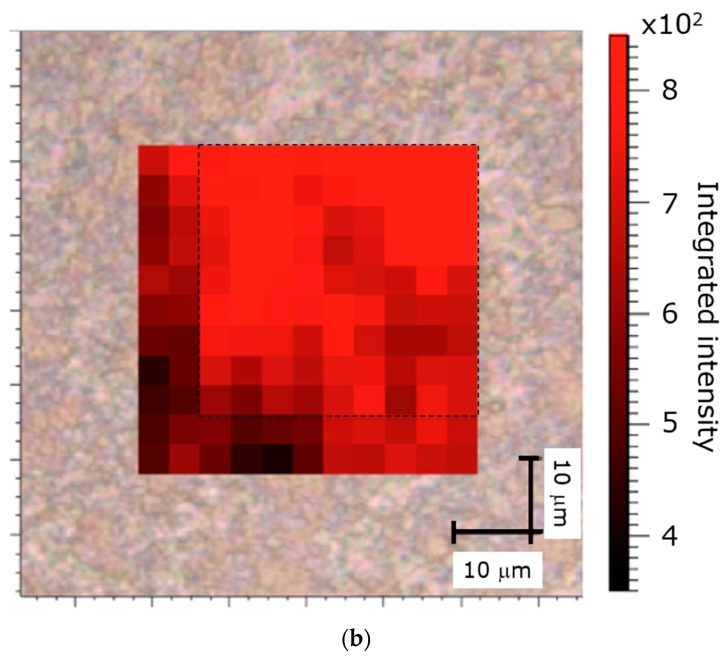
(**a**) Raman spectra of benzenethiol (BZT) adsorbed over nanowires (black line) and over evaporated gold surface (dark yellow) compared to the DFT calculations: generalized gradient approximations (GGA) approximation (red line) and LDA(Local Density Approximation) (blue line). The inset shows the absence of Raman signal coming from the bare device. In this case, the laser power was fixed to 10 mW and acquisition time of 1 s. In the side panels, the main vibrational modes are reported; (**b**) SERS 2D mapping of the Raman band centered around 1572 cm^−1^ of BZT deposited over Au nanowires, showing the rather uniform distribution of the signal throughout the large area of the device. The laser power and integration time were fixed to 1.25 mW and 1 s. The average intensity and the standard deviation σ in the whole area is Ῑ_whole_ = (7.0 ± 1.4) × 10^2^. In the inner dashed subarea Ῑ_sub_ = (7.6 ± 1.0) × 10^2^.

To verify the device performance, we have physisorbed R6G and chemisorbed BZT over the SERS surfaces. BZT (10^−4^ M) and R6G (10^−5^ M) solution were prepared in ethanol and water solvent, respectively. SERS spectrum of BZT was excited through 100×, NA = 0.9, objective with the laser power 0.5 mW (to avoid any specimen modification due to laser power) and the acquisition time was fixed to 20 s. SERS spectrum of BZT is shown in Figure 4a. Various characteristic vibrational Raman bands of BZT can be observed centered at around 998, 1021, 1073, and 1572 cm^−1^, which may be attributed to the breathing mode of the benzene ring, ring deformation mode, C–S stretching, and ring stretching vibration, respectively [33,34], besides, many other broad peaks populate the spectral region. A reference spectrum of BZT deposited over flat Au surface measured with the same laser illumination power is shown in Figure 4a. Background Raman measurement, in the same figure, clearly indicates that the SERS substrate was free from any impurities. Furthermore, a SERS 2D-mapping measurement was also carried out in an area of around 40 × 40 µm^2^, as shown in Figure 4b. We show the intensity map for the Raman band centered at 1572 cm^−1^. The result shows a quite uniform Raman signal from all the active area of SERS substrate. As quantitative metrics, we measured the average intensity and the standard deviation σ for the whole area of Figure 4b, Ῑ_whole_ = (7.0 ± 1.4) × 10^2^, and the intensity for a more uniform subarea (the inner region delimited by the dashed lines in Figure 4b) where Ῑ_sub_ = (7.6 ± 1.0) × 10^2^.

Two simulations based on density functional theory (DFT) were performed for BZT molecule attached to a gold atom, Figure 4a right panels. The calculations were performed using ADF2016 package. The molecule was firstly optimized in geometry and, then, Raman spectra were estimated using generalized gradient approximations (GGA) of Perdew–Burke–Emzerhof (PBE), and LDA (Local Density Approximation) exchange functional and triple-zeta polarization (TZP) basis set. We show in Figure 4a left the experimental (black line) and theoretically calculated (red and blue lines) Raman spectra. 

It must be pointed out that the simulation of Raman spectra of molecules chemisorbed on SERS substrates is pretty complex: while DFT calculations are very effective in calculating the Raman spectra of free molecules in solutions, the chemisorption, on a noble metal surface, forces the molecule to be arranged in different configurations, with local charge transfer that depends on the topographical details of the metal surface and on the electric field enhancement. Consequently, the Raman response presents strong modification of the experimental spectra respect to free molecule. 

These substantial changes in scattering spectra depend specifically on each different substrate [35,36,37,38]. DFT Modeling of this interaction is not straightforward because of the large number of metallic atoms required to properly account the presence of a surface bond and the distortion induced by field enhancement. Nevertheless, DFT shows that SERS spectra can be different from Raman in liquid phase, but remains still a useful tool for understanding the effect of the substrate. In any case, simulations help to unambiguously assign the different bands to the corresponding vibrational modes of the molecule.

To get the description of the different modes of the molecules, we have therefore employed, in this work, a simplified model in which the molecule is bound to a single gold atom attached to the thiol group, which already allow us to assign the observed peaks to specific vibrations of the molecule (main vibrational modes shown as short movies in supporting material). A detailed study considering a cluster of gold atoms attached to the thiolated molecule will be discussed in a next publication.

**Figure 5:**
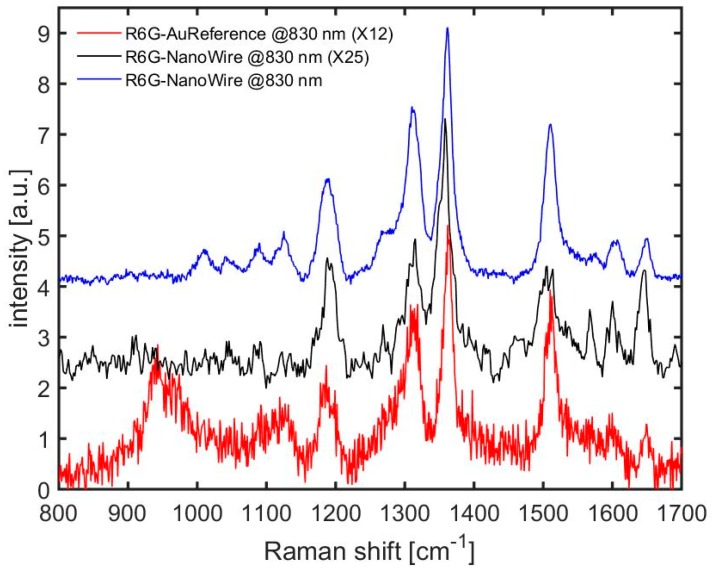
Raman spectra of R6G excited at 830 nm (6 mW, 1 s) and 633 nm (2 mW, 1 s). Notice the black curve is multiplied by a factor 25, for sake of comparison. The spectrum of R6G on evaporated gold reference at 830 nm excitation wavelength is also reported multiplied by a factor 12 (red curve). An additional offset is added for better presentation.

Further, we measured the Raman spectrum of R6G using 633 and 830 nm laser excitation to test the device for the use with a fluorescent molecule. In this case, the interaction of the molecules with the metal leads to a physical absorption, so that a single monolayer cannot be obtained, differently from BZT, where a monolayer of molecules is covalently chemisorbed on gold. Figure 5 shows the resulting spectra as obtained after subtraction of the fluorescent background. The main peaks centered at 1180, 1310, 1360, 1510, and 1650 cm^−1^ can be related to the C–H_x_ bending, combination of C–H and N–H bending, combination of ring C–C stretching and N–H bending, combination of C–N stretching and C–H bending, and combination of ring stretching C–C vibration and C–H bending, respectively [39]. SERS enhancement in intensity is clearly observed with respect to the reference evaporated Au surface. We observed a lower Raman intensity when we investigate the sample with 633 nm with respect to the 830 nm. This is because the highest field enhancement is in the NIR region, as can be observed in Figure 3 and Figure S1. 

## 3. Discussion

In this work, we have proposed a new type of SERS device. By using numerical simulations, it is possible to optimize the structure for best performance in a broadband centered at the desired excitation wavelength, keeping in mind that our fabrication technique is largely flexible in controlling the geometrical parameters. To this purpose, we have simulated many device configurations by varying the gap between nanowires, the Au height, and the nanowire diameter, as shown in Figure S1b. In all simulations, the gold wires have been considered partially embedded in an alumina matrix, in order to be the closest possible to the real situation of our sample.

For the specific case, relevant for the comparison with our experimental results, we have simulated a SERS substrate with pillar diameter equal to 97 nm and height equal to 400 nm, in order to estimate the electric field distribution, and then to compare it with the experimental measurements. The detailed information about the simulation configurations is summarized in the Materials and Methods section. 

The simulations show that the confinement of electric field remains strong in the whole spectral range from 550 to the NIR; in particular, the electric field enhancement reaches a factor about 14 for 830 nm, whereas it reaches, at best, around 10 when excited with 633 nm laser. The three different polarization directions simulated demonstrate that the electric field depends weakly on the incident polarization directions, and that the field enhancement patterns remain stable for all configurations used, in virtue of the packed trigonal symmetry of the structure and of the circular shape of each single wire. As expected, the top edges of the wires show the highest enhancement along the interwire axis, which is characterized by a specific spectral shape, as shown in Supplementary Information Figure S1a–e. In these figures, we report the spectral electric field amplitude at different positions within the gaps as a function of the field polarization direction, and for different vertical distances below and above the nanowire tops. Finally, results of FDTD simulations on a single Au/Fe-wire identify the single-wire contribution to the total device field enhancement. In particular, the peak at about 570 nm identifies the LSPR of the single vertical nanowire and, in fact, it appears in both types of simulation, as expected. Further details are reported in the Supplementary Information.

Regarding the experiments, we performed a series of SERS measurements at 830 nm using BZT, which shows a large increase in the local intensity with respect to the reference measurement. We estimated the enhancement factor for the nanowire substrate to be in the order of 10^4^ with respect to the flat Au reference. It must be noted that this value might appear relatively lower with respect to the best results shown in the literature, but a few things can be taken into account: these values are calculated with respect to gold surface, which already shows its own enhancement (reported to be in the order of 10 [40,41]. Figure 4b shows the low variability of the signal from an extended active area across the sample. Finally, the device is broadband, and the enhancement can be obtained in a large portion of the spectrum, while many resonant systems are much more wavelength sensitive, and show best enhancement in a narrower spectral region.

As a second experimental test, we collected Raman spectra of R6G excited by 830 and 633 nm laser lines, which are shown in Figure 5 for two different samples.

As a last consideration, we comment about the reproducibility and reliability of the device regarding the micro-Raman technique. In particular, considering a wire interdistance d = 100 nm in the trigonal structure, we can evaluate the laser spot dimension, w_0_, as the diffraction limit beam waist for a fixed wavelength and focusing objective: w_0_ = 2λ/(πNA) ~ 0.7λ. With these assumptions, we can calculate the expected hot spots concurrently active during the single measure to be about $\frac{\pi }{2}\left(\frac{w_{0}}{d}\right)\left(\frac{w_{0}}{d\sqrt{3}/2}\right)$ for the polarization along one of the trigonal sides, and nearly the double in the vertical polarization case, Figure 3. For the 830 nm excitation, this means a minimum number of hot spots from about 64 to 128, a number that averages out, sensibly, the variability observed in the typical SERS measurement based on single hot spot devices.

We can estimate the experimental average field enhancement per gap unit at 830 nm considering a hot spot diameter of 2 nm, as the ratio of the area and the interested number of hot spots within the focal point ($\mathrm{beam}\;\mathrm{waist}\;w_{0})$. Considering the measure reported in Figure 4, evaluating a ratio about 12.6 between SERS and reference spectra, and that $\left(w_{0}{}^{2}\right)/\left(2^{2}\right)/64\;≃1318$, we estimated a local signal Raman enhancement of about 1.6 × 10^4^, corresponding, at first approximation order, to a field amplitude boost of ~11 with respect to the incidence laser field. This value is close to the average hot spot value obtained from the FDTD simulation.

In conclusion, these results demonstrate the viability of the present device as SERS substrate in the vis–NIR region, with potential applications in the detection of several molecules, ranging from simple chemicals to molecules of biological interest. By varying the geometrical parameters, localized and collective plasmon resonances of the system can be tuned across the visible to NIR region, while maintaining a large enhancement in this wavelength range. Finally, the device is weakly sensitive to polarization orientation. Since no sophisticated instruments were employed to build these nanostructures, the device can be considered a large area cost-effective plasmonic sensor for chemical analysis.

## 4. Materials and Methods

### 4.1. Fabrication of Nanowire from Porous Alumina Template

Highly ordered hexagonal porous alumina template were fabricated by anodization process optimized by ref. [27]. Pure aluminum disks (99% purity and 500 nm thickness) were purchased from *GoodFellow*(Huntingdon, UK). The disks, after cleaning with acetone and isopropanol, were electropolished in a bath of perchloric acid and ethanol [HClO_4_/C_2_H_6_O] (% *v*/*v*) = 1/4 with the bias voltage of 20 V for 3 min. We used the disk as an anode, and a platinum grid as cathode. A custom Teflon cell (KAUST lab, Thuwal, SA) was used for a 2-step anodization process of the aluminum disk. The first anodization run was at a constant voltage of 40 V for 24 h in 0.3 M oxalic acid solution at a temperature of 4 °C. Aluminum disk was only anodized to the front face. To achieve this, the disk was fixed between a copper plate and the Teflon cell. The hole through the Teflon allows the contact with the bath, while an O-ring avoids leakage of the solution. Then, the solution was stirred near the anode at 300 rpm. After that, the porous alumina film was etched away in an acidic chrome solution (CrO_3_/H_3_PO_4_ in water) overnight at 40 °C. Finally, a second anodization under the same conditions created the ordered APA template. The aluminum underneath was then removed with an acidic copper chloride solution. Drops of 5% H_3_PO_4_ in water (*w*/*w*) were applied to the surface for 2 h to etch away the nanofilm of alumina covering the channels. An anodic porous alumina (APA) is finally attained. In the last step, the porous alumina templates were dipped in 5% H_3_PO_4_ in water (*w*/*w*) to widen the pores.

APA templates were used to grow metal nanorods through the pores by electrodeposition. A gold film of 200 nm was sputtered on the porous alumina template surface and, thereafter, firstly iron and then gold were deposited. The bath for gold electroplating was made of 0.1 g of potassium dicyanoaurate (KAu(CN)_2_ from Sigma-Aldrich, St. Louis, MO, USA), 4 g of boric acid (H_3_BO_3_ from Fisher, Hampton, NH, USA) in 100 mL of H_2_O, while the bath for the iron electroplating was made of 6 g of iron sulfate heptahydrate (FeSO_4_·7H_2_O from Fisher), 1 g of boric acid, and 1 g of ascorbic acid. The fabrication process is shown in Figure 1. 

### 4.2. Optical Characterizations

We used a Renishaw InVia Raman spectrometer with a 1200 lines/mm grating for the SERS measurements. The samples were excited by 633 and 830 nm laser lines in backscattering configuration through 100× objective (NA = 0.9) using the respective edge filters to stop the laser lines. The scattering was collected in the whole range 200–2000 cm^−1^. The spectra were analyzed with WiRE 3. The reference measurements were made on 50 nm gold-coated Si substrate. Rhodamine 6G and benzenethiol were deposited on the substrates by immersion in solution and subsequent rinsing in MilliQ water. 

### 4.3. Electromagnetic Simulations

The simulations of the device were carried out using a commercial software package performing finite-difference time-domain calculations (FDTD Solutions, Lumerical Inc., Vancouver, CA, USA). A plane wave pulse in the range of 400–1000 nm illuminated the multiwires device from the top, or the single vertical wire. Periodic boundary conditions were chosen to simulate an infinitively extended sample. The FDTD simulation used an adaptive mesh strategy, refining the mesh around the nanowire to a fixed value of 0.5 nm in the plane of the sample, and of 1 nm in the perpendicular direction. The diameter of the nanowire and the gap were set, respectively, to 97 nm and 6 nm. We placed detection monitors at nine positions along the vertical axis (−6, −3, −1, 0, +1, 2, +3, +6, +12 nm from the pillars top), to describe the near field around the top surface plane of Au wires, as shown in Figure S1a–g. With these intensity profiles, we evaluated the field confinement in the horizontal plane, as well as the vertical confinement between the pillars and above the device. 

## Supplementary Materials

The following are available online at http://www.mdpi.com/1996-1944/11/5/827/s1. Supplementary information reports the FDTD analysis and the graphical representation of all the electric field distributions considered in the text. It also reports examples of different nanowires based devices we fabricated. Five short movies show the main vibration modes considered for the BZT molecule.
