# Supplementary material for: Plasmonic Nanowires for Wide Wavelength Range Molecular Sensing
Source: Materials (Basel). 2018 May 17;11(5):827. doi: 10.3390/ma11050827 (PMC5978204; doi:10.3390/ma11050827)
Supplement: Supplementary file 1 [file materials-11-00827-s001.zip › materials-291852 SI-rev2.pdf]

Supplementary Information

Plasmonic nanowires for wide wavelength range molecular sensing.

G. Marinaro [1], G. Das [2], A. Giugni[2], M. Allione[2], B. Torre[2], P. Candeloro[3], J. Kosel[1] and E. Di Fabrizio[2,*]

[1] King Abdullah University of Science and Technology (KAUST), 4700 Kingdom of Saudi Arabia – Division of computer, electrical and mathematical sciences and engineering (CEMSE);
[2] King Abdullah University of Science and Technology (KAUST), 4700 Kingdom of Saudi Arabia – PSE Division, SMILEs lab.
[3] BioNEM Lab. Department of Experimental and Clinical Medicine. University Magna Graecia, Catanzaro 88100, Italy
* Correspondence: enzo.difabrizio@kaust.edu.sa

Sections:

1. Calculation of electric field distribution
2. Fabrication of different nanowires based device

1. Calculation of electric field distribution:

Electric field (EF) distribution was calculated using FDTD. The devices were excited using three polarization configurations (horizontal, vertical and 60° from horizontal). Periodic conditions were chosen in X- and Y-axis considering the possibility to represent the trigonal unitary cell of the template within a larger rectangular cell. The calculation was performed in the range between 400-1000 nm with the mesh resolution, refined around the Au/Fe wires, of 0.5 nm in X- and Y- directions and 1 nm in Z-direction.
Figures S1(a-c) show the EF distribution at different height above and below the wires top plane (from -6 nm to +12 nm) for five significative wavelengths (830, 785, 633, 570, and 405 nm), for the tree above mentioned polarizations. In all the cases, the strongest hot spots are visible at 830 nm and gets weaker moving toward the visible. The simulations at 0° and 60° of polarization show equivalent results, as expected due to the trigonal symmetry of the sample, thus confirming the correctness of the chosen periodic boundaries.
Figure S1d shows the spectral intensity of the electric field at different positions from the center of the gap between two nanowires toward the center of the wire, as indicated in the bottom-right panel. Each panel reports curves taken at different heights above and below the wire top, to evaluate the extinction and the confinement of the field around the structure top surface.
The EF spectral profile at Z=0 shows a broad enhancement region having its maximum in NIR, that extends in the visible down to about 570nm, due to the presence of the single wire resonance aside the periodicity effect, as shown in figure S1d-g. The spectrum of a single wire was obtained from a specific simulation, considering a wire embedded in the alumina substrate, as shown in Figure S1h left. In this case we used perfect matching layers boundaries in the simulation instead of infinite periodic ones. Figure S1h right, reports the comparison of the reflectance and transmittance spectra obtained for three different structures: the SERS device, the simple alumina film and the single wire.
We also performed FDTD simulations to understand the effect of the gap width on the device. Figure S1i reports spectra obtained at the edge of the wire and at the center of the gap for structures having different gap width and same periodicity. As the figure shows, it is clear the highest field enhancement is observed in the structure having the narrower gap, where the enhancement also remain high for all the broadband range from 570 nm down to the NIR.

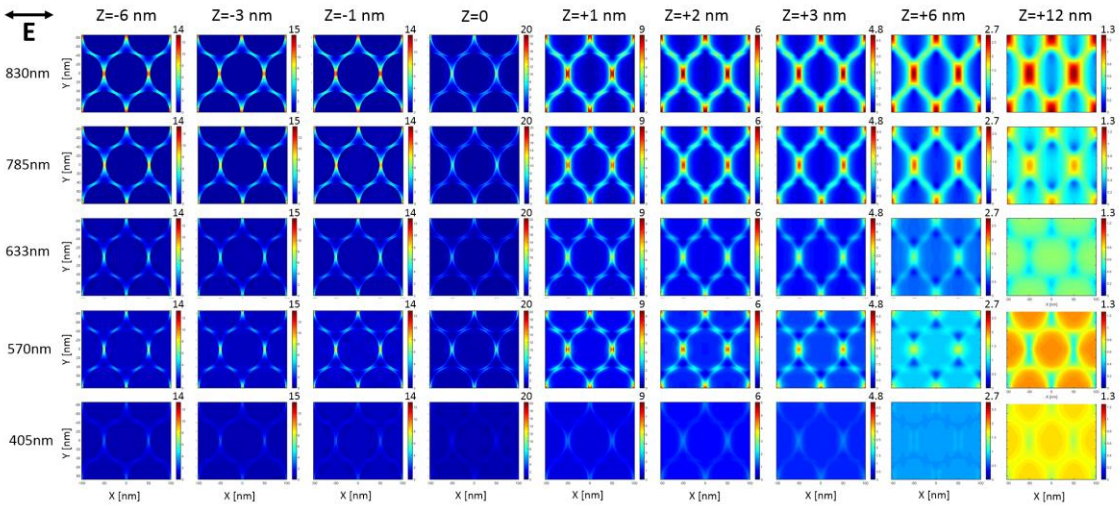

Figure S1a: Electromagnetic FDTD simulations result for the electric field amplitude distribution obtained at 405, 570, 633, 785 and 830 nm when the device is excited with plane wave polarized along the short wires inter-axes (horizontal). Each column represents a XY plane (200 x180 nm) at the indicated fixed Z quote. The intensity color map has been normalized column by column to get maximal visibility of the field distribution.

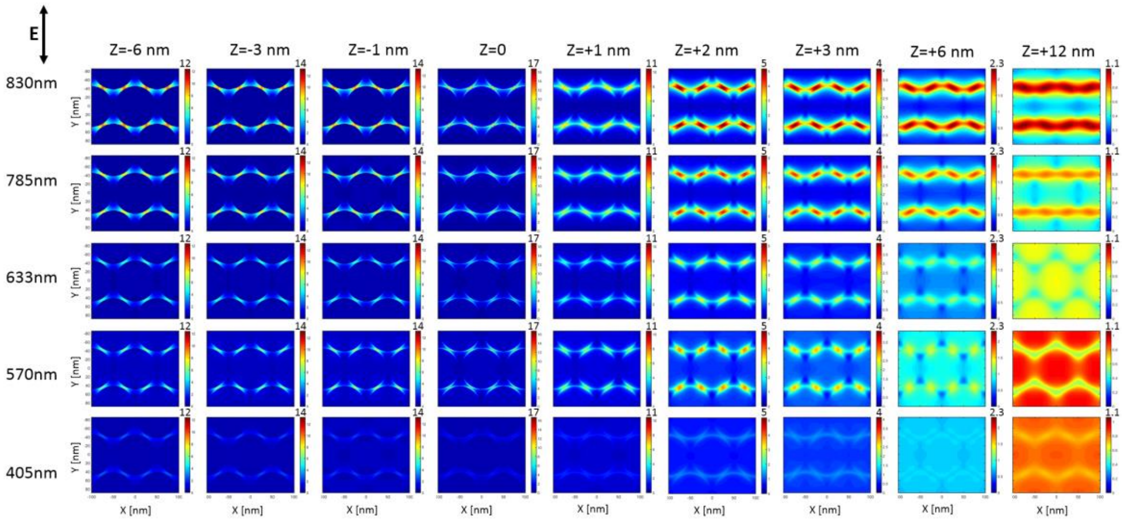

Figure S1b: Electromagnetic FDTD simulations result for the electric field amplitude distribution obtained at 405, 570, 633, 785 and 830 nm when the device is excited with plane wave polarized along the short wires inter-axes (vertical). Each column represents a XY plane (200 x180 nm) at the indicated fixed Z quote. The intensity color map has been normalized column by column to get maximal visibility of the field distribution.

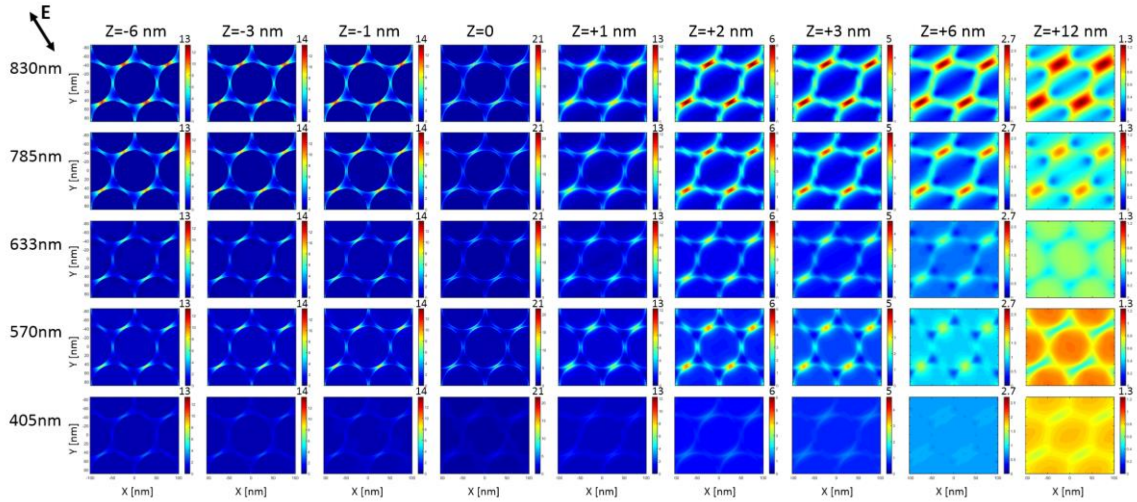

Figure S1c: Electromagnetic FDTD simulations result for the electric field amplitude distribution obtained at 405, 570, 633, 785 and 830 nm when the device is excited with plane wave polarize 60° with respect to the short wires inter-axes (horizontal). Each column represents a XY plane (200 x180 nm) at the indicated fixed Z quote. The intensity color map has been normalized column by column to get maximal visibility of the field distribution. This configuration is equivalent to the one of Figure S1a.

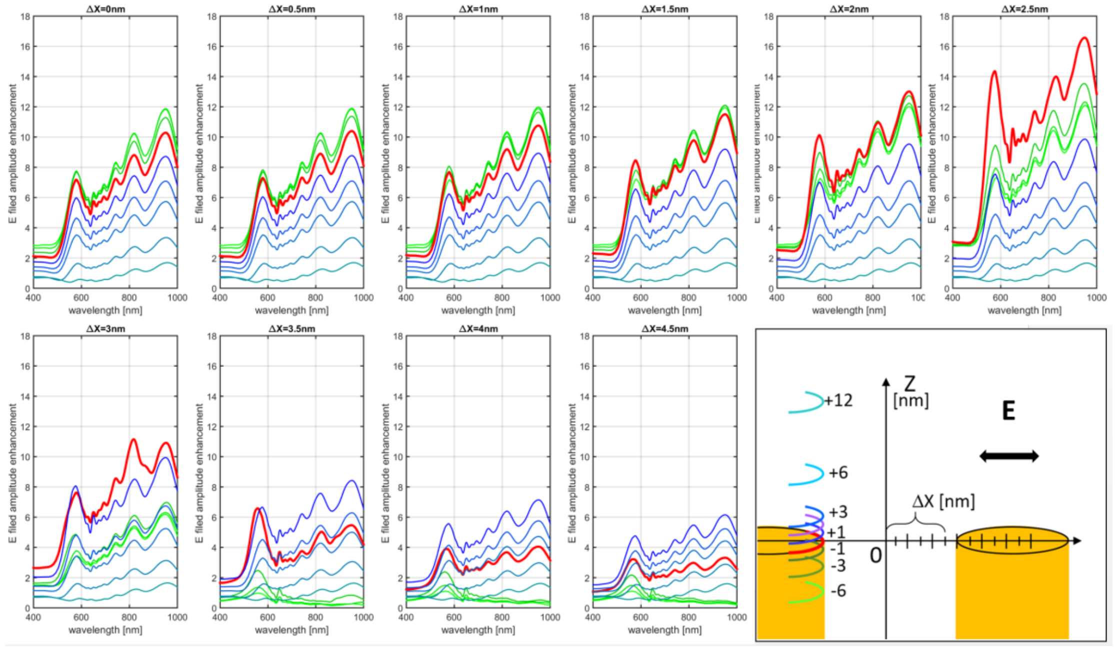

Figure S1d: Electromagnetic FDTD simulations result for the spectral amplitude of the electric field when excited with plane wave polarized along the short wires inter-axes (horizontal) and normal incidence, in function of the position, Δx, along the gap axis (as shown in the scheme). Each graph reports curves obtained at different Z quote, as indicated in figure with a colors assignation.

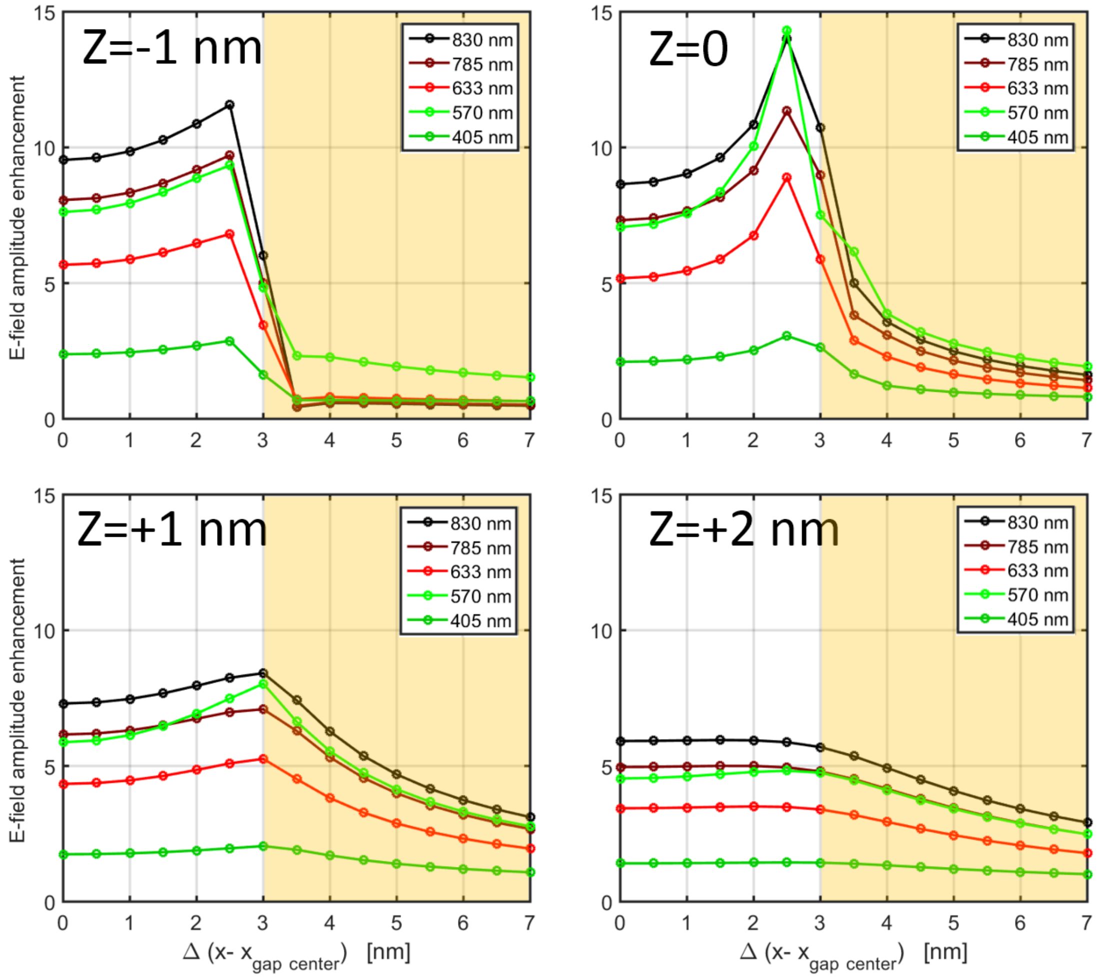

Figure S1e: Electromagnetic FDTD simulations results. Amplitude of the electric field for five representative wavelengths as a function of the inter-gap distance, Δx when excited with plane wave polarized along the short wires inter-axes (horizontal) and normal incidence. Each graph reports curves obtained at different Z quote around the top wire, as indicated.

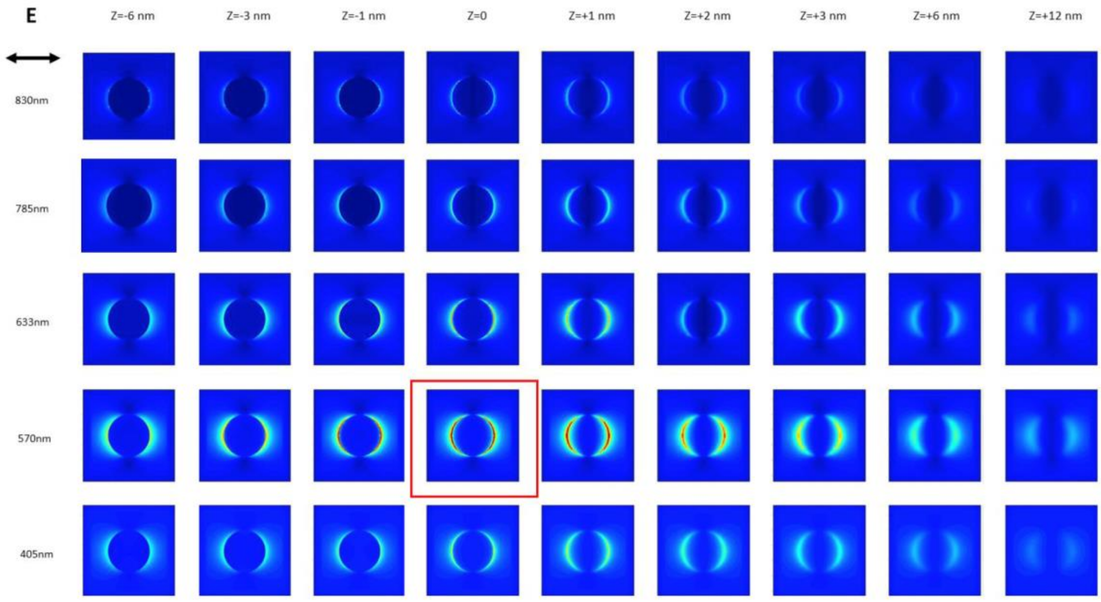

Figure S1f: Electromagnetic FDTD simulations result for the electric field amplitude distribution obtained at 405, 570, 633, 785 and 830 nm when a single wire is excited with plane wave polarized along the horizontal axis and with normal incidence. Each column represents a XY plane (200 x180 nm) at the indicated fixed Z quote. The intensity color map has been normalized to the same value for all the panels to get maximal visibility of the absolute maximum of the electric field distribution (the maximum enhancement obtained was about 10-time, for 570 nm at Z=0).

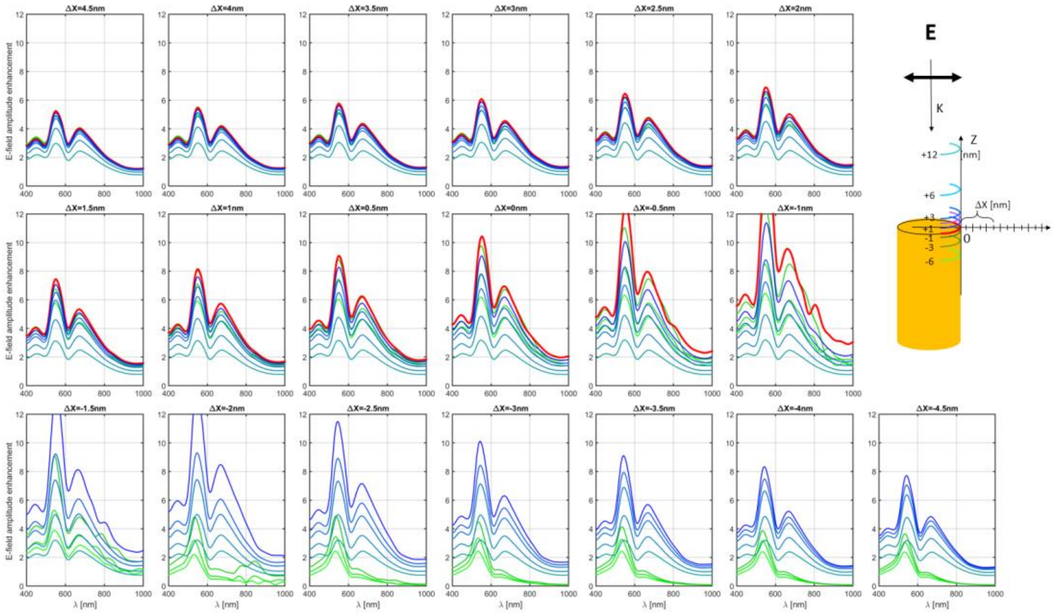

Figure S1g: Electromagnetic FDTD simulations results for the spectral amplitude of the electric field when a single wire is excited with plane wave polarized along the horizontal axis and with normal incidence, in function of the position Δx along the polarization axis (as indicated in the scheme). Each graph reports curves obtained at different Z quote, as indicated in figure with a colors assignation.

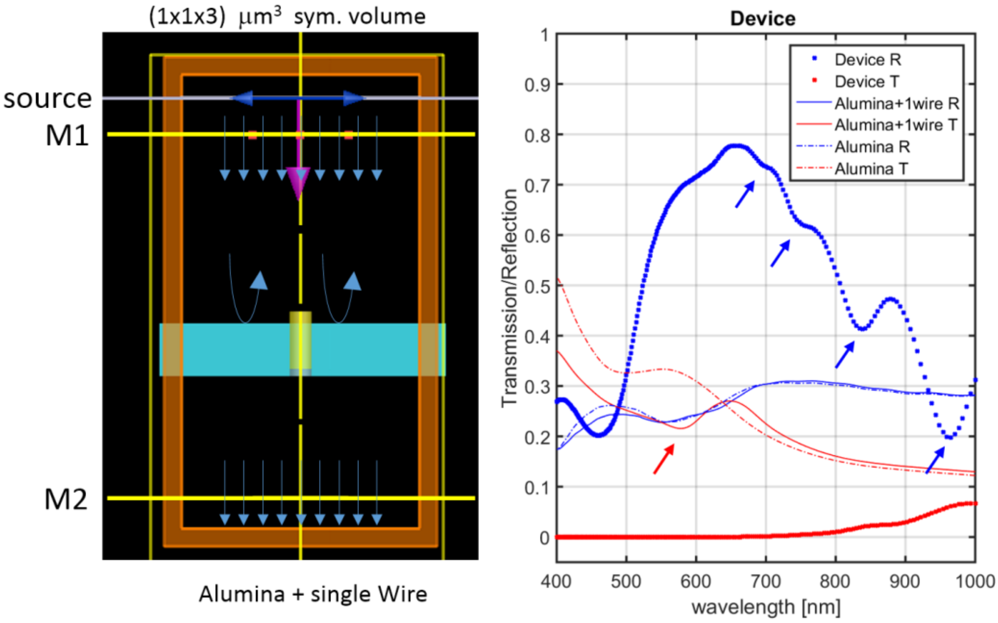

Figure S1h: Left, schematic representation of the simulated environment for a single wire embedded in alumina matrix. The two monitors, M1 and M2, used to

evaluate the reflectance and transmittance of the devices are indicated. Right, transmittance and reflectance of three different cases: SERS device, device with a single standing wire, and alumina thin film. Blue arrows evidence the effect of periodicity, while the red one indicate the contribution of single pillar, comparing to the simple alumina layer.

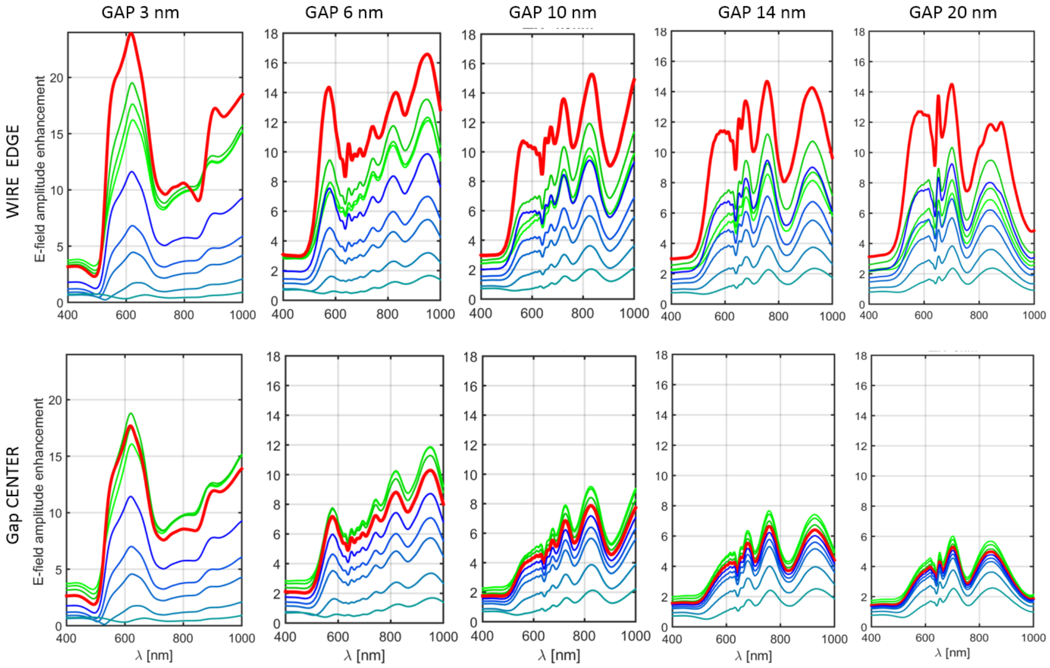

Figure S1i: Electromagnetic FDTD simulations results. Spectral amplitude of the electric field of the SERS device excited with plane wave polarized along the horizontal axis and with normal incidence, at the gap center and at the wire edge, for structures with different gaps. Each graph reports curves obtained at different Z quote, according to the same color code of Figure S1g.

2. Fabrication of different nanowires based device: Figure S2 shows SEM pictures of devices at different steps of the fabrication process. These pictures demonstrate the versatility of the fabrication of APA substrate, which allows to control the pore size and the wall thickness, and allows obtaining different structural parameters in the final fabricated nanowires structure.

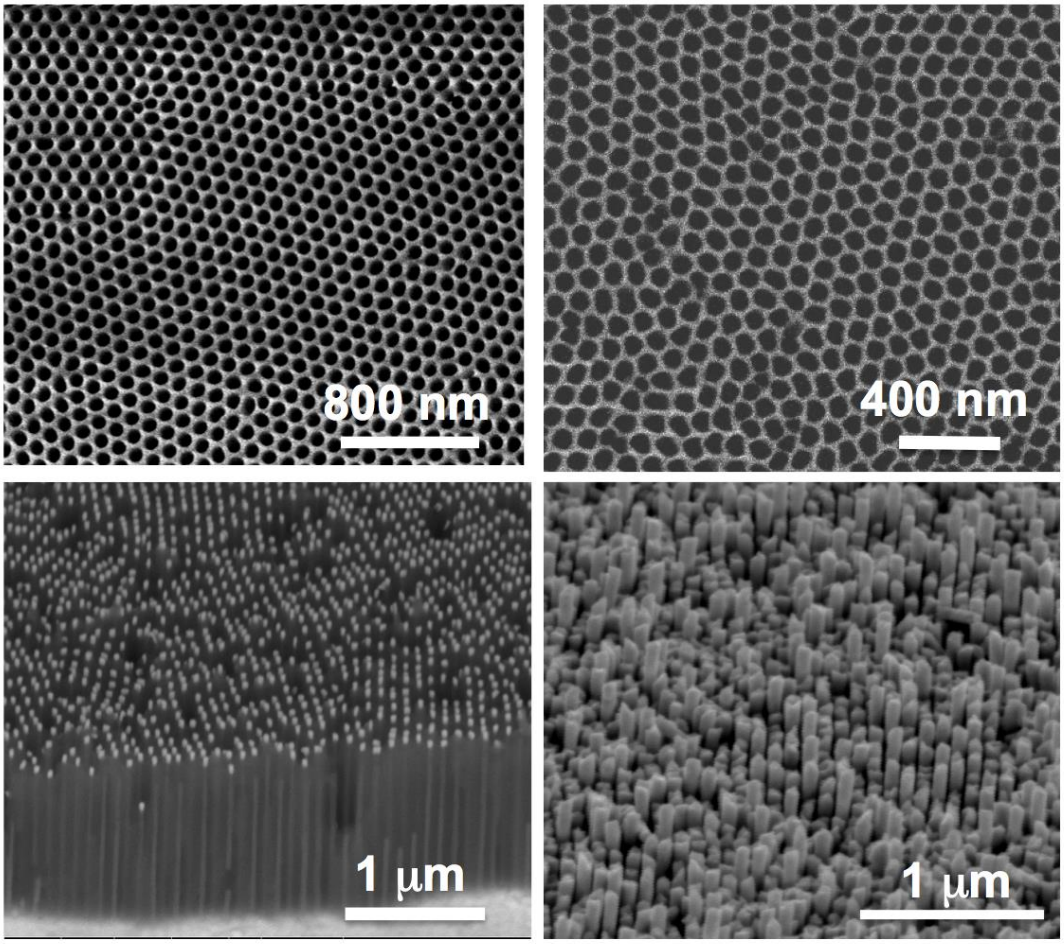

Figure S2: SEM images of samples at different steps of the fabrication process: top, images of APA templates for two different diameters. Bottom, side view of different final devices, obtained with different pore sizes and changing the thickness of the two metals used thus tuning the length of the Au/Fe wire obtained.
